# Low survival in younger adults with Acute Myeloid Leukemia (AML) in Tanzania linked to high disease burden and socioeconomic factors

**DOI:** 10.1371/journal.pone.0332237

**Published:** 2025-09-19

**Authors:** Michelle Munroe, Clara Chamba, Mbonea Yonazi, Samira Mahfudh, William Mawalla, Kelvin Mbelekwa, Gladys Kaaya, Rebbecca Mwakichako, Johnson Mshiu, Magdalena Lyimo, Mwashungi Ally, Ahlam Nasser

**Affiliations:** 1 Department of Hematology and Blood Transfusion, Muhimbili University of Health and Allied Sciences, Dar es Salaam, Tanzania; 2 Ministry of Health, Georgetown, Guyana; 3 Department of Hematology, Georgetown Public Hospital and Corporation, Georgetown, Guyana; 4 Department of Hematology, Muhimbili National Hospital, Dar es Salaam, Tanzania; 5 Department of Clinical Oncology, Muhimbili University of Health and Allied Sciences, Dar es Salaam, Tanzania; 6 Department of Oncology, New Amsterdam Regional Hospital, Berbice, Guyana; 7 Department of Hematology, Ocean Road Cancer Institute, Dar es Salaam, Tanzania; 8 Department of Hematology, Moi County Referral Hospital, Taita-Taveta, Kenya; 9 National Institute for Medical Research, Muhimbili Research Center, Dar es Salaam, Tanzania; Stanford University, UNITED STATES OF AMERICA

## Abstract

**Background:**

Acute Myeloid Leukemia (AML) accounts for 20–25% of all leukemia diagnosed worldwide. According to Globoccan 2020, leukemia ranked the 15^th^ most prevalent cancer with an estimated 474,519 new cases and 311,594 deaths annually. However, due to scarcity of well documented cancer registries, epidemiological and survival data of patients with AML is lacking in many African countries, including Tanzania. Therefore, the primary objective of this study was to determine the clinical features, laboratory characteristics and survival outcomes of AML patients treated with different regimens in Tanzania.

**Methods:**

Data from all adult patients diagnosed with AML at Muhimbili National Hospital (MNH) between January 2018 and December 2023 were analyzed in this retrospective study. STATA version 16 was used for data analysis. The survival outcome was calculated using the Kaplan-Meier method. To evaluate the statistical significance of the results, a p-value cut-off of 0.05 was used. The Cox proportional hazards model was used to identify predictors of survival and to estimate the effect of covariates on the hazard of death. Kruskal Wallis was used to compare the median values for laboratory results and pairwise comparison of median laboratory values was done using the Dunn Test.

**Results:**

A total of 245 patients were diagnosed with acute leukemia during the five-year period (2018−2023), of which 169 (68%) had AML. The mean age at diagnosis was 47.2 (SD ± 18.5) years. Majority of the patients were females (60.7%), and had de novo AML. Most of the patients presented with symptoms of anemia (98.2%) and fever (79.5%). The median survival time was 81 days with a one-year overall survival probability of 15.2%. Patients that resided outside of the Dar es Salaam region had a 74% increase hazard of mortality compared to those living within the Dar es Salaam region (aHR: 1.74, 95% CI: 1.15–2.64, p = 0.008). Patients receiving any form of chemotherapy had a 39% lower hazard of mortality compared to those who were on supportive treatment alone (aHR: 0.61, 95% CI: 0.40–0.93, p = 0.022).

**Conclusion:**

AML is the most common acute leukemia among adults in Tanzania, primarily affecting young females under 50 years. Resource constraints and limited treatment options result in poor outcomes, with a median survival of 81 days and one-year overall survival of 15.2%.

## Introduction

Acute myeloid leukemia (AML) is a type of cancer that originates in the bone marrow and is characterized by a rapid proliferation of immature myeloid cells, known as blasts. This uncontrolled growth leads to a significant reduction in the production of normal blood cells, resulting in conditions such as anemia, thrombocytopenia, and neutropenia [1]. The disease progresses quickly, often requiring immediate treatment to prevent severe complications, including life-threatening infections and bleeding. In the United States (US), there are around 20,000 new cases of AML cases diagnosed every year. The mean age at the time of diagnosis is around 65 years [1]. In the United Kingdom (UK), 42% new cases of AML are diagnosed in persons older than 75 years [2]. Leukemia was the 10th most prevalent cancer type in sub-Saharan Africa in 2018, with an estimated 20,900 new cases and 17,600 deaths annually [3]. Due to unavailability of nationwide cancer registries in many African countries, data on the incidence of AML and other hematological malignancies remain scarce. However, single-institution studies have reported the proportion of AML ranging from 4% to 20% of all hematologic malignancies. For instance, a study conducted at the Uganda Cancer Institute reported AML accounting for approximately 8.8% of hematologic malignancies [4]. These variations may reflect differences in diagnostic capacity, referral patterns, and population demographics across regions. Notably, the mean age at diagnosis in Africa has been reported to be nearly two decades lower than in Caucasian populations. For example, previous studies done in Kenya and Tanzania have reported a mean age at diagnosis between 35 and 40 years. [5_,_^6^].

Despite the relatively younger patient population observed in African countries, the survival outcomes for patients with AML remain poor, largely due to limited resources, including diagnostic facilities, access to chemotherapy, and supportive care. In Kenya, Wanjiku et al. reported a one-year survival rate of only 7.9% for patients with non-M3 AML, with a median overall survival of 45 days following diagnosis [5]. Similar findings were observed in Uganda, where the one-year survival was 16.5%, and the median survival time was 47 days [7].

Observations from Tanzania are not much different from other East African countries. A study on Pediatric Cancers by Schroeder et al demonstrated that among patients who received intensive chemotherapy, 84% of those with acute lymphoblastic leukemia (ALL) and 35% of those with acute myeloid leukemia (AML) achieved complete remission [8]. However, long-term outcomes remained poor. The estimated 2-year event-free survival (EFS) was 33% for ALL patients, whereas AML patients had an EFS of 0% [8], this underscores that the challenges of managing AML are beyond availability of intensive chemotherapy. Reliable and consistent access to supportive care including blood and product transfusion, isolation units for infection prevention and broad spectrum anti-microbials are among the challenges commonly faced in many resource limited settings like Tanzania [9].

According to the current international standard treatment protocols, the choice of treatment for adult patient with AML largely depends on the fitness of the patient to withstand the intensity of the regimen [10]. The less fit patients are typically treated with less intensive regimens such as hypomethylating agents (HMA), supportive care, or may be enrolled in clinical trials. Whereas fit patients undergo European Leukemia Net (ELN) risk stratification to classify them as favorable, intermediate, or unfavorable risk. Favorable risk patients are treated with induction and consolidation chemotherapy, intermediate risk may receive consolidation chemotherapy or hematopoietic cell transplantation (HCT), while unfavorable risk benefit from allogeneic HCT following remission after induction chemotherapy [11].

The majority of adult AML patients in Tanzania receive low-dose cytarabine with palliative intent, and only a small number undergo induction therapy. Although standard induction and consolidation chemotherapy agents are available in the country, affordability remains a significant barrier for many patients. In addition, the reliable and consistent supply of blood and blood products to meet transfusion demands during treatment poses another critical challenge. Given the limited access to comprehensive and continuous supportive care, palliative regimens remain the mainstay of treatment. Assessing survival outcomes under the current treatment approaches is essential to establish baseline data that can create strategies aimed at improving AML care and survival in Tanzania.

## Materials and methods

### Study design, area, and population

This retrospective review was conducted at the Muhimbili National Hospital (MNH) in Dar es Salaam and included a data review of medical records of all patients diagnosed with AML by bone marrow aspiration morphology from March 2018 to December 2023.

### Selection criteria

#### Inclusion criteria.

Patients aged 18 years and above, diagnosed with Acute myeloid leukemia by bone marrow aspiration morphology from March 2018 to December 2023.

#### Exclusion criteria.

Patients with Acute Promyelocytic Leukemia (APML), classified as French-American-British (FAB) M3, those with Chronic Myeloid Leukemia (CML) in blast crisis, and those with incomplete data insufficient for analysis were excluded. APML and CML in blast crisis were excluded to maintain homogeneity of the study population, as their treatment approaches differ from those of other AML subtypes.

### Enrollment of participants and data collection.

Data was collected from the digital hospital record system (JEEVA) and patients’ paper files, recorded on a predefined data collection sheet and then entered into a Microsoft Excel sheet.

### Data analysis

STATA version 16 was used for data analysis. Descriptive statistics like percentage and mean/median were employed to describe the patients’ demographic, clinical, and laboratory characteristics. Kruskal-Wallis test was done to compare the median values and interquartile ranges for laboratory results. The Dunn Test was used to demonstrate a pairwise comparison of median laboratory values. The survival outcome was calculated using the Kaplan-Meier method. To evaluate the statistical significance of the results, a p-value cut-off of 0.05 was used. The Cox proportional hazards model was used to determine predictors of survival and calculate the effect of covariates on survival.

### Ethical clearance

Ethical clearance was obtained from two primary bodies, Muhimbili University of Health and Allied Science (MUHAS) Institutional Review Board (IRB) and MNH Research and Consultancy Bureau. Data was kept anonymous and was only used for this study. The IRB waived the requirement for informed consent. The approval code is: MUHAS-REC-2023-1998.

## Results

### Frequency of acute myeloid leukemia

**Fig 1:**
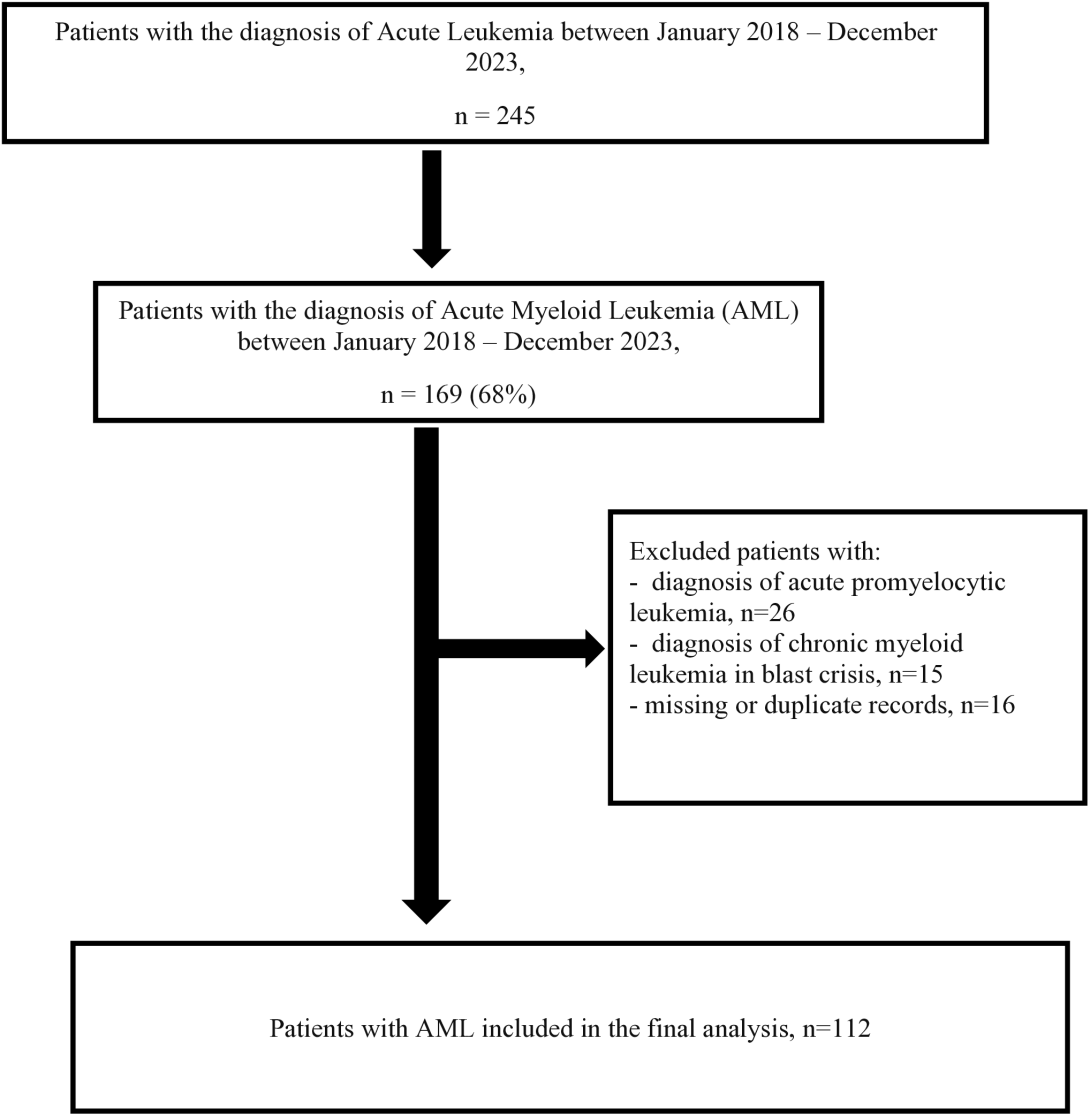
Flow chart of acute leukemia cases. Flow chart showing 245 patients were diagnosed with Acute Leukemia between January 2018 and December 2023. Of these, 169 patients (68%) had Acute Myeloid Leukemia (AML). A total of 112 patients with AML were included in the final analysis.

A total of 245 patients were diagnosed with acute leukemia during the five-year period (2018–2023), of whom 169 (68%) had AML. Of these, 57 were excluded from further analysis: 26 had APML, 15 had CML in blast crisis, and 16 were missing/duplicate records. The remaining 112 patients were included in the final analysis (Fig 1).

### Sociodemographic and clinical characteristics of the study participants

Out of the 112 patients diagnosed with AML, the majority of the patients, 68 (60.7%) were females, with a female to male ratio of 1.5:1. One-half of the patients, 56 (50.4%) were below 49 years, with the mean age at diagnosis of 47.2 (± 18.5) years. Nearly half of the patients, 52 (46.4%) resided outside Dar es Salaam where MNH, the center with hematology services is located. Only one-third of the patients, 31(27.7%) had health insurance covering all or part of their medical bills. Most of the patients were considered fit, with a small portion of 20 (17.9%) presenting with co-morbidities Table 1.

**Table 1:** Social demographic and clinical characteristics of the patients (n = 112).

Variable	Frequency	Percentage
**Sex**		
Male	44	39.3
Female	68	60.7
**Age group**		
Below 29	26	23.4
30-49	30	27.0
Above 49	55	49.6
**Place of residence**		
Dar es Salaam	60	53.6
Outside Dar es Salaam	52	46.4
**Insurance status**		
Insured	31	27.7
Not insured	81	72.3
**Comorbidities** *		
No	92	82.1
Yes	20	17.9
**De novo AML**		
No	8	7.1
Yes	103	92.0
Unspecified	1	0.9
**Secondary AML**		
No	103	92.0
Yes**	8	7.1
Unspecified	1	0.9

*Diabetes mellitus, Hypertension, Human immunodeficiency virus (HIV), Hepatitis B, Hepatitis C

**Therapy related AML cases were4

**Fig 2:**
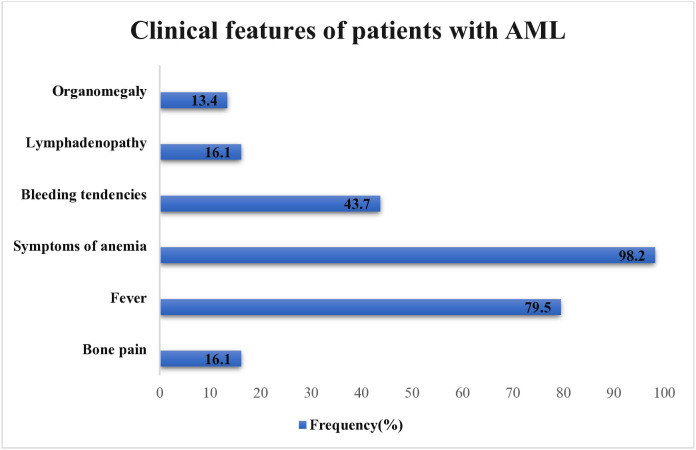
Clinical features of patients with acute myeloid leukemia. Bar chart showing the frequency of clinical features at diagnosis in patients with Acute Myeloid Leukemia (AML). Symptoms of anemia (98.2%), fever (79.5%) and bleeding tendencies (43.7%) were the most common clinical features.

The most common presenting clinical features of the patients were symptoms of anemia, 110 (98.2%), fever,89 (79.5%), and bleeding tendencies, 49 (43.7%). Other presenting features, such as bone pain and lymphadenopathy, were occasionally observed in 18 patients (16.1%), while organomegaly was unexpectedly present in 15 patients (13.4%) (Fig 2).

### Treatment regimens and laboratory characteristics of patients with AML

**Fig 3:**
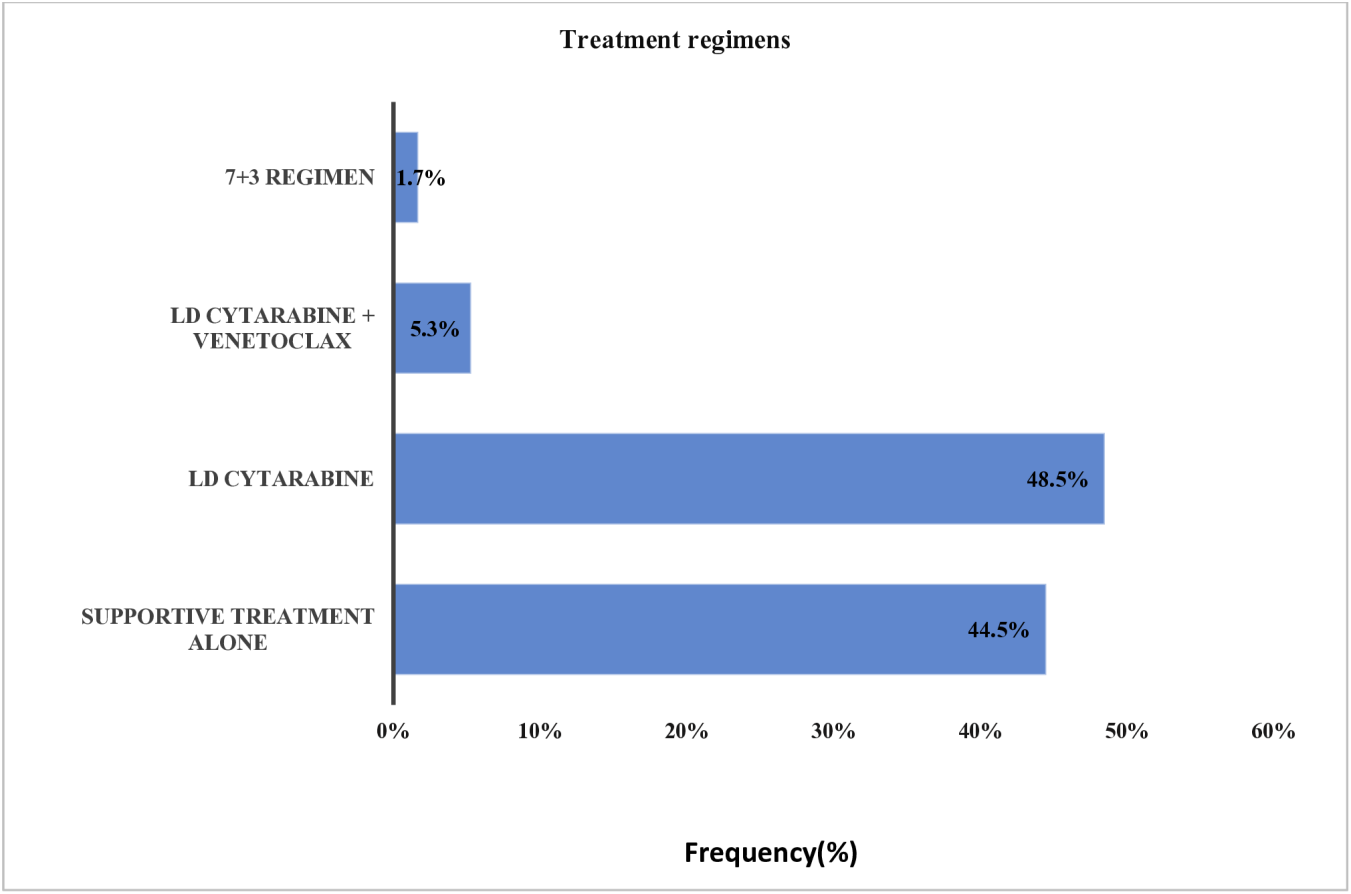
Treatments regimens used by patients with non-M3 AML. Bar chart showing the proportion of patients who received different treatment regimens for Acute Myeloid Leukemia. The majority were treated with low-dose cytarabine (48.5%) or supportive care alone (44.5%), both administered with palliative intent.

The majority of patients, 54 (48.5%), received low-dose cytarabine alone, followed by 50 (44.5%) who received supportive care only. Only 6 patients (5.3%) were able to afford low-dose cytarabine plus venetoclax, while 2 patients (1.7%) could afford the intensive 7 + 3 chemotherapy regimen (7 days of cytarabine and 3 days of daunorubicin or idarubicin) (Fig 3).

### Median Blood counts at diagnosis and after receiving the first and fourth treatment cycles of low-dose cytarabine or low-dose cytarabine with venetoclax

There was a significant improvement in blood counts observed after the first and fourth treatment cycles. The median white blood cell (WBC) count declined from 14.9 × 10⁹/L (IQR: 5.5–51.3) at diagnosis to 4.3 × 10⁹/L (IQR: 2.8–5.6) after the fourth treatment cycle (p < 0.001). Similarly, significant increases in median hemoglobin (Hb) and platelet levels were observed after the fourth treatment cycle compared to baseline levels at diagnosis Table 2.

**Table 2:** Median values and IQR for laboratory results.

Variable	Stage			
	At diagnosis Median (IQR)	After first cycle Median (IQR)	After fourth cycle Median (IQR)	*p-value
WBC (x 10^9^/L)	14.9(5.5–51.3)	3.6(1.8–15.2)	4.3(2.8–5.6)	<0.001
ANC (x 10^9^/L)	1.8(0.4–6.4)	0.5(0.1–1.6)	0.7(0.2–2.0)	0.005
HB (g/dl)	6.1(5.1–7.3)	7.4(5.6–8.6)	10.1(8.9–11.6)	<0.001
PLT (x 10^9^/L)	35.6(18.5–58.6)	45(20.5–82.8)	197.0(84.6–245.0)	0.001

*Kruskal Wallis

**KEY**

WBC: White blood cell count

HB: Hemoglobin

ANC: Absolute neutrophil count

PLT: Absolute platelet count

**Fig 4:**
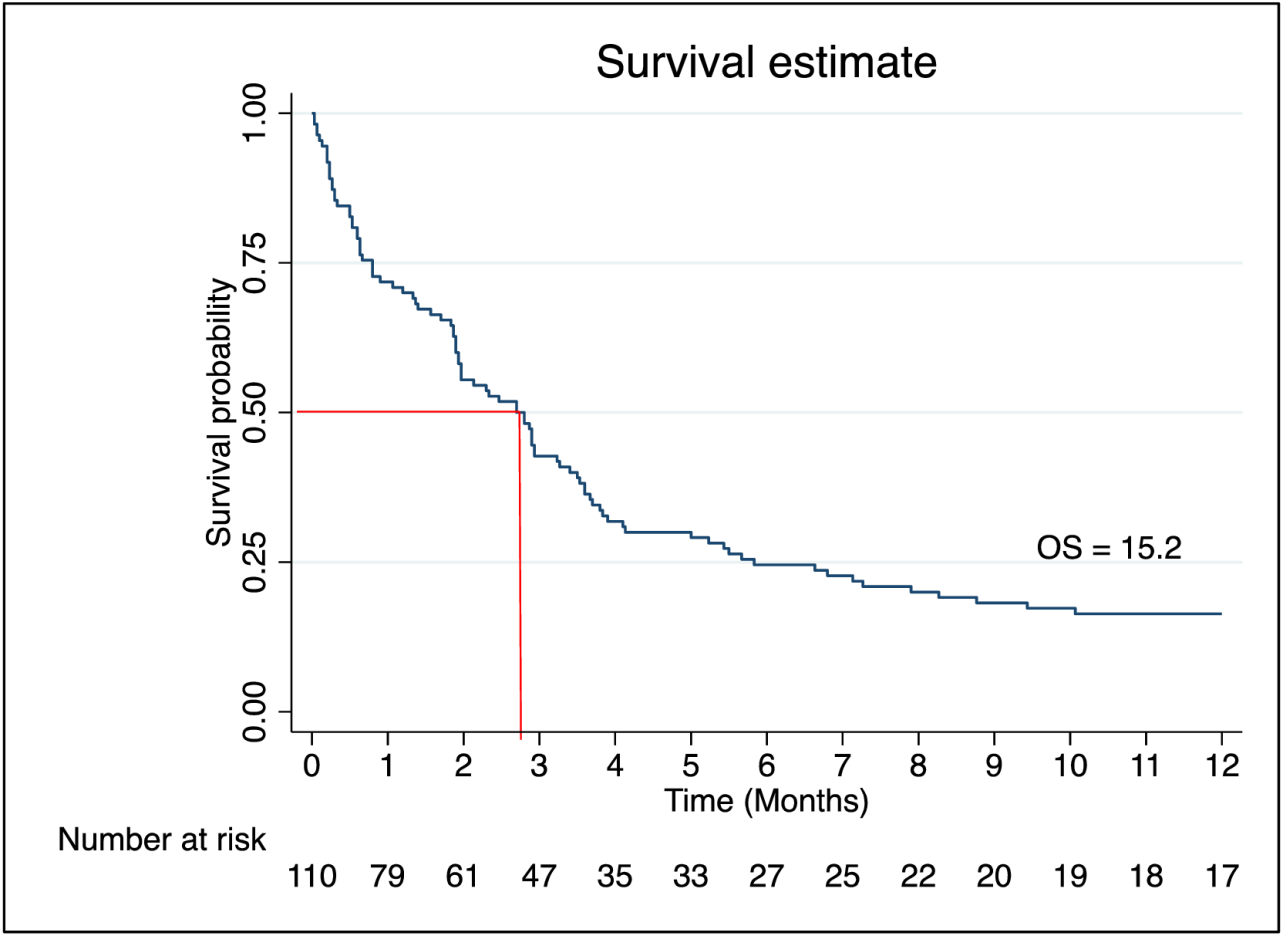
Kaplan-Meier curve showing overall survival of non-M3 acute myeloid leukemia patients in one year.

**Fig 5:**
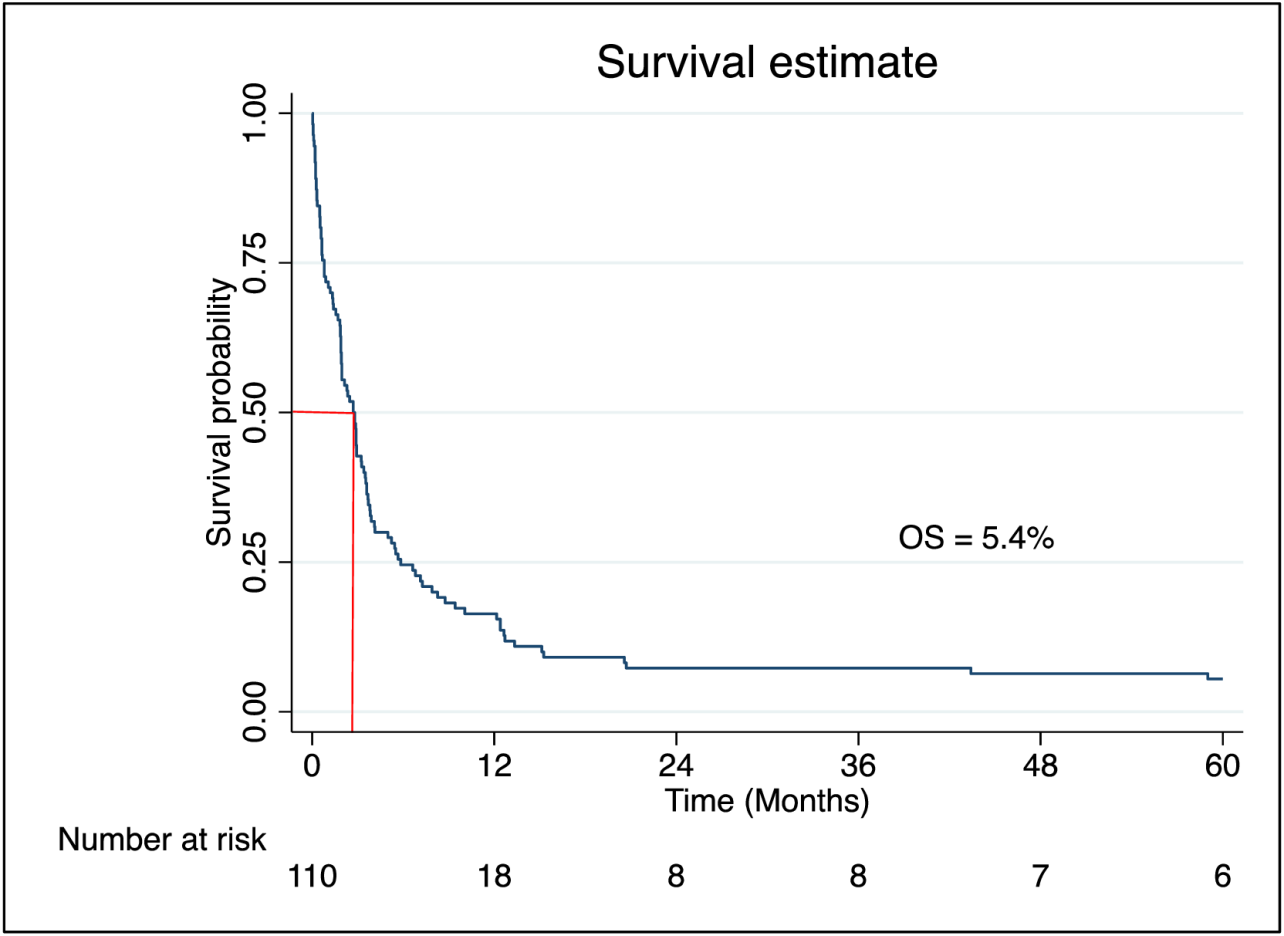
Kaplan Meier curve showing the five-year overall survival among patients with non-M3 acute myeloid leukemia.

**Fig 6:**
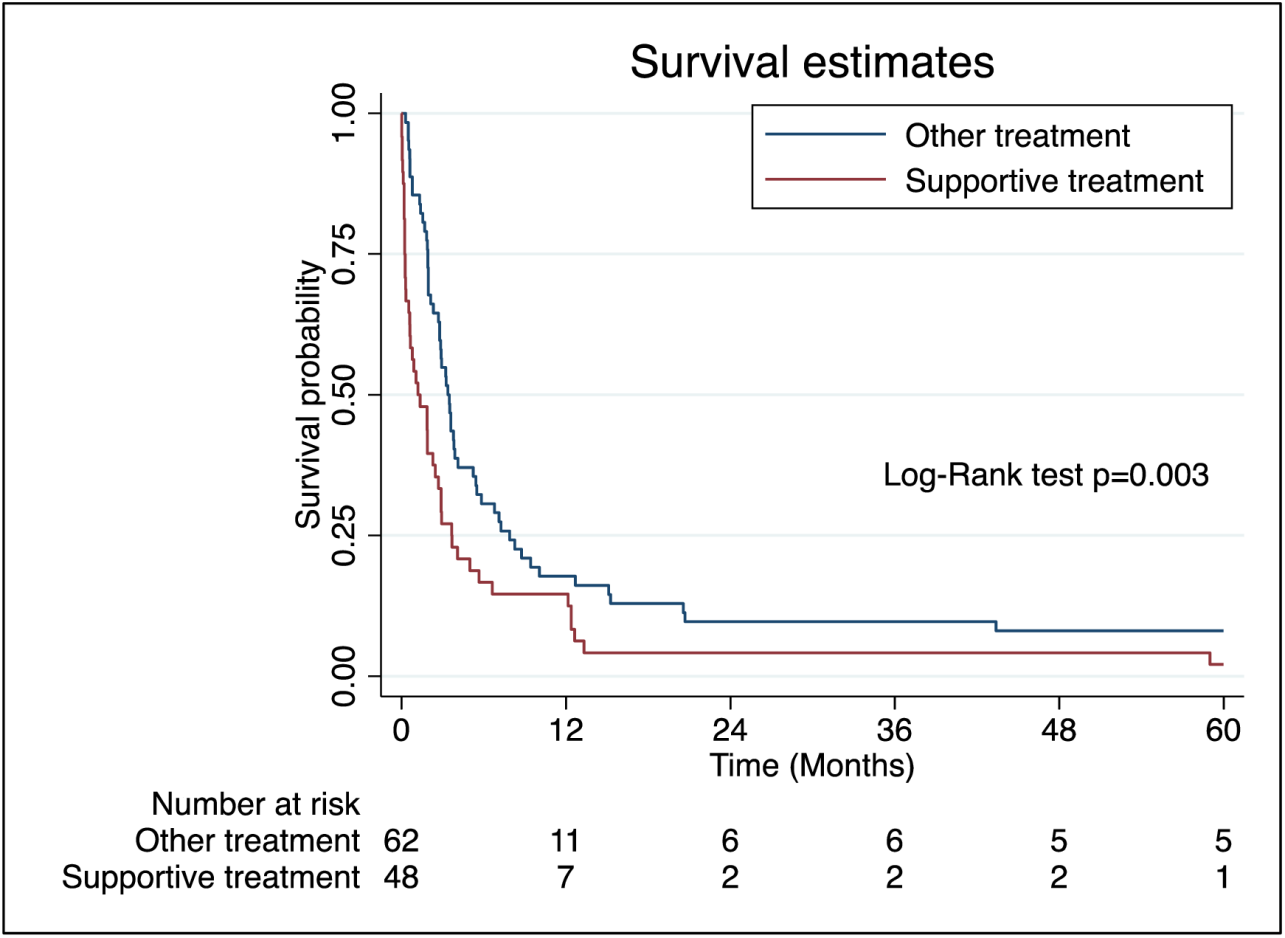
Log Rank Test for AML patients on supportive care alone versus AML patients on treatment with LD cytarabine/LD cytarabine + venetoclax or intensive chemotherapy in addition to supportive care. Kaplan–Meier curves demonstrate higher overall survival among patients with Acute Myeloid Leukemia (AML) who received any form of treatment (low-dose cytarabine, low-dose cytarabine plus venetoclax, or intensive chemotherapy) compared to those who received supportive care alone (Log-rank test, *p* = 0.003).

Patients receiving low-dose (LD) cytarabine or LD cytarabine with venetoclax had median survival times of 98 and 105 days respectively, while those on supportive care alone had a significantly shorter median survival of 36 days Table 3. Overall median survival for the cohort was 81 days. Survival probability at 90 days was 42.0% (95% CI: 32.8–50.9), dropping to 24.1% at 180 days, 17.9% at 270 days, and 15.2% at one year (Fig 4). At the end of five-year, the overall survival (OS) for non-M3 AML was 5.4% (Fig 5). When comparing patients receiving any form of treatment (LD cytarabine or LD cytarabine + venetoclax or intensive chemotherapy) compared to supportive care alone, the OS was higher for patients on any form of treatment compared to supportive care alone (Log-rank test, *p* = 0.003) (Fig 6).

**Table 3:** Median survival time for acute myeloid leukemia.

Variable	Number of patients	Survival time		
		25%	50%	75%
Supportive treatment alone	50	7	36	110
LD Cytarabine	54	57	98	214
LD Cytarabine with Venetoclax	6	84	105	263
Total	110	24	81	175

*LD: Low Dose*

### Factors associated with the survival of patients with acute myeloid leukemia

In univariate analysis, residing outside Dar es Salaam was associated with a 67% higher risk of mortality (cHR = 1.67, 95% CI: 1.12–2.49), while treatment with low-dose cytarabine was linked to a 40% reduction in mortality compared to supportive care alone (cHR = 0.60, 95% CI: 0.41–0.90). Multivariate analysis confirmed these associations: patients living outside Dar es Salaam had a 74% higher mortality risk (aHR = 1.74, 95% CI: 1.15–2.64, *p* = 0.008), and those treated with low-dose cytarabine compared to supportive care alone had a 39% lower mortality risk (aHR = 0.61, 95% CI: 0.40–0.93, *p* = 0.022). Table 4.

**Table 4:** Cox regression for factors associated with the Hazard of death among patients with Acute Myeloid Leukemia.

Variable	cHR (95% CI)	*p-value*	aHR (95% CI)	*p-*value
**Sex**				
Female	Ref		Ref	
Male	0.94(0.63-1.39)	0.755	0.95(0.64-1.42)	0.817
**Age group**				
Below 29	Ref		Ref	
30-49	1.41(0.81-2.45)	0.230	1.27(0.72-2.26)	0.408
Above 49	1.19(0.72-1.96)	0.491	1.38(0.81-2.35)	0.240
**Place of residence**				
Dar es Salaam	Ref		Ref	
Outside Dar es Salaam	1.67(1.12-2.49)	0.011	1.74(1.15-2.64)	**0.008**
**Insurance status**				
Insured	Ref		Ref	
Not insured	1.35(0.88-2.07)	0.176	1.23(0.73-2.07)	0.430
**Comorbidities**				
No	Ref		Ref	
Yes	0.87(0.53-1.44)	0.595	0.94(0.52-1.71)	0.851
**Treatment regimens**				
Supportive treatment	Ref		Ref	
LD cytarabine	0.60(0.41-0.90)	0.013	0.61(0.40-0.93)	**0.022**
LD cytarabine and venetoclax	0.43(0.17-1.08)	0.071	0.51(0.23-1.11)	0.090
7 + 3 regimen	0.20(0.03-1.49)	0.118	0.28(0.03-2.99)	0.289

## Discussion

### Frequency and baseline characteristics of patients diagnosed with AML

The frequency of AML among adult patients diagnosed with acute leukemia over a 5-year period (2018–2023) was 68%. This is similar to other studies done in East Africa. A study by Natukunda et al in Uganda showed a prevalence of AML of 58.4% among patients diagnosed with acute leukemias [7]. Another study done in Nepal, showed 46.7% of patients with acute leukemia had AML [12].

In our study, the majority of AML patients were below 49 years, with a mean age at diagnosis of 47.2 years which is significantly younger than what is typically reported in high-income countries [13–16]. This likely reflects the younger demographic profile of low and middle income countries (LMICs) [5,7], but may also point to region-specific risk factors beyond aging, such as genetic predisposition, environmental exposures including infections (particularly viral infections), socioeconomic barriers, and limited access to early healthcare [17]. Delayed diagnosis of precursor conditions like MDS may also contribute [18–20]. This younger age distribution underscores the need for localized research to better understand etiological factors associated with AML and other hematological malignancies.

AML is generally reported to be slightly more prevalent in males than females [21,22]. However, in our study, we observed a higher number of female patients compared to males. This female predominance in our cohort may be attributed to differences in health-seeking behavior between genders and the demographic profile of our population. According to the 2022 census data, Tanzania’s population includes slightly more females than males, with 33.13 million females compared to 32.37 million males [23]. In contrast, studies from other regions have reported male predominance; such as in Uganda, 59.2% of AML cases were male, while in Nepal, 71.7% of those diagnosed were male [7,11].

### Clinical features and laboratory characteristics of patients diagnosed with AML

The majority of our patients presented with symptoms of anemia and fever, while nearly half exhibited bleeding tendencies. These findings reflect bone marrow dysfunction due to uncontrolled blast proliferation, which impairs the production of other hematopoietic cell lines. The complete blood count at diagnosis showed severe anemia and thrombocytopenia, consistent with the clinical presentation. However, the white blood cell count demonstrated leukocytosis, with a median (IQR) of 14.9 (5.5–51.3) ×10⁹/L. These findings are consistent with those reported in previous studies [24,25].

### Treatment and survival outcome

With the advent of new treatment approaches, overall survival for AML patients has significantly improved in high-income settings [26,27]. Among young and fit adults with favorable-risk cytogenetics, the one-year overall survival (OS) can exceed 65%. Although this rate is lower in patients with unfavorable-risk cytogenetics, the one-year OS still typically exceeds 50% [28].

In LMICs such as Tanzania, optimal AML treatment is often hindered by multiple systemic barriers. These include the lack of advanced diagnostic platforms for accurate risk stratification, limited availability or affordability of intensive chemotherapy, targeted therapies, and bone marrow transplant services [29,30]. Additionally, optimal supportive care comprising of blood and blood product transfusions, appropriate isolation facilities, and access to a broad range of antimicrobials is a critical and necessary component of AML management, without it, mortality rates remain unacceptably high [31–33]. However, access to these resources remains inconsistent, particularly where transfusion services are constrained. Given these limitations, low-dose cytarabine is often the mainstay therapy for adult AML patients in Tanzania, even for those eligible for intensive treatment. In our study, most patients received low-dose cytarabine with palliative intent, however it showed significant improvement in blood counts between the first and fourth cycles. This underscores the benefit of any chemotherapy compared to supportive care alone, although responses are typically short-lived. As expected, the one-year overall survival (OS) was low at 15.2%, with a median survival of 81 days. This was comparable to the 16.4% reported in Uganda, although their median survival was slightly lower at 55 days [7]. These poor outcomes among adult patients in LMICs are especially troubling, given that many of these individuals are young and physically fit, and could otherwise tolerate intensive chemotherapy. Importantly, they often serve as primary breadwinners for their families and represent a vital segment of the national workforce. This underscores the urgent need for countries like Tanzania to invest in improving the affordability and accessibility of cancer care in order to achieve better survival outcomes.

In our cohort, among the factors that were associated with increased mortality, apart from being on supportive care alone, included residing outside of Dar es Salaam which further highlights the increasing resource constraints especially transfusion services outside of Dar es salaam, where the main National Referral Hospital is located.

These findings emphasize the need for policy interventions to expand critical healthcare services, including health insurance coverage, and to decentralize cancer care beyond major urban centers in Tanzania. While the government’s efforts to introduce advanced treatments such as hematopoietic cell transplantation (HCT) [34] to reduce the need for seeking care abroad are commendable, concurrent strengthening of basic services such as transfusion, chemotherapy access, and a broad range of antimicrobials, is essential to improve overall patient outcomes. Universal health insurance coverage is a key political strategy and holds great potential to improve access to care; however, ensuring that essential treatments, including commonly used chemotherapies must be prioritized. Our study demonstrated that despite insurance coverage, treatment modalities did not differ significantly, as most targeted therapies, immunotherapies, and basic chemotherapies are currently not covered by health insurance schemes.

### Study strength and limitations

One strength of this study is that it is the first in Tanzania to report on survival outcomes of adult patients with AML. However, it was a single-center study with a relatively small sample size, which may limit the generalizability of the findings. Additionally, the lack of immunophenotyping may have led to underestimation of AML cases, particularly in instances where undifferentiated or minimally differentiated blasts may have been misclassified as Acute Lymphoblastic Leukemia (ALL) based on morphology alone.

## Conclusion

Acute Myeloid Leukemia (AML) is the most common type of acute leukemia among adults in Tanzania, with the majority of patients being young females under the age of 50 years. Due to resource limitations, low-dose cytarabine remains the mainstay of treatment, resulting in a reduced median survival time of only 81 days and a one-year overall survival of 15.2%. These findings highlight the urgent need for strategies to improve access to effective treatment and supportive care for AML patients in Tanzania.
